# Associating cryptogenic ischemic stroke in the young with cardiovascular risk factor phenotypes

**DOI:** 10.1038/s41598-020-79499-1

**Published:** 2021-01-11

**Authors:** Joseph M. Dardick, David Flomenbaum, Daniel L. Labovitz, Natalie Cheng, Ava L. Liberman, Charles Esenwa

**Affiliations:** 1 Department of Neurology, Albert Einstein College of Medicine, 3316 Rochambeau Avenue, Bronx, NY 10467 USA; 2 Stern Stroke Center, Montefiore Medical Center, Bronx, NY USA

**Keywords:** Stroke, Neuro-vascular interactions, Neurological disorders, Risk factors

## Abstract

Acute Ischemic Stroke (AIS) in the young is increasing in prevalence and the largest subtype within this cohort is cryptogenic. To curb this trend, new ways of defining cryptogenic stroke and associated risk factors are needed. We aimed to gain insights into the presence or absence of cardiovascular risk factors in cases of cryptogenic stroke. We conducted a retrospective cohort study of patients aged 18–49 who presented to an urban tertiary care center with AIS. We manually collected predefined demographic, clinical, laboratory and radiological variables. Clinical risk phenotypes were determined using these variables through multivariate analysis of patients with the small and large vessel disease subtypes (vascular phenotype) and cardioembolic subtype (cardiac phenotype). The resultant phenotype models were applied to cases deemed cryptogenic. Within the 449 patients who met criteria, patients with small and large vessel disease (vascular phenotype) had higher rates of hypertension, intracranial atherosclerosis, and diabetes mellitus, and higher admission glucose, HbA1c, admission blood pressure, and cholesterol compared to the patients with cardioembolic AIS. The cardioembolic subgroup (cardiac phenotype) had significantly higher rates of congestive heart failure (CHF), rheumatic heart disease, atrial fibrillation, clotting disorders, left ventricular hypertrophy, larger left atrial sizes, lower ejection fractions, and higher B-type natriuretic peptide and troponin levels. Adjusted multivariate analysis produced six variables independently associated with the vascular phenotype (age, male sex, hemoglobin A1c, ejection fraction (EF), low-density lipoprotein (LDL) cholesterol, and family history of AIS) and five independently associated with the cardiac phenotype (age, female sex, decreased EF, CHF, and absence of intracranial atherosclerosis). Applying these models to cryptogenic stroke cases yielded that 51.5% fit the vascular phenotype and 3.1% fit the cardiac phenotype. In our cohort, half of young patients with cryptogenic stroke fit the risk factor phenotype of small and large vessel strokes.

## Introduction

While progress has been made in decreasing the overall mortality associated with acute ischemic stroke (AIS) over the last half-century, the incidence of AIS is climbing^1^. This is especially true among the young, where rates of AIS have increased by as much as 91% in the past 15 years^2^. The long-term impacts of AIS in the young are more severe than in older patients due to the number of years lived with increased disability and risk of recurrence^3^. These patients also have higher age-matched mortality when compared to the general population^4^.

It is hypothesized that the climbing rate of AIS in the young is driven by a paralleled premature rise in traditional cardiovascular disease (CVD) risk factors^2,5^. The prevalence of these within this cohort, however, is still far below that of older patients and a recent study found that AIS in the young is not completely matched by the increased prevalence of traditional risk factors^2^. This suggests that there may be additional under-recognized vascular or non-vascular risk factors accounting for a higher than usual proportion of cryptogenic stroke in the young.

We sought to identify what proportion of stroke risk in young patients with cryptogenic AIS could be attributed to traditional CVD risk factors, as seen in large artery and small vessel “lacunar” strokes, versus possible cardiac disease^6^. Using relevant clinical, biological, and radiographic markers from a cohort of young patients with AIS, we defined phenotypic profiles of three determined stroke subtypes (small vessel [“lacunar”], large artery atherosclerosis [LAA], and cardioembolic [CE]). We then used these profiles to characterize patients in the same study cohort with the cryptogenic strokes as either having (1) a combined lacunar/LAA, termed “vascular,” phenotype, (2) a CE, termed “cardiac,” phenotype, or (3) neither, to gain insight into the possible CVD drivers of cryptogenic stroke in the young.

## Patients and methods

### Study design

We conducted a retrospective cohort study of consecutive patients with AIS aged 18–49 admitted to either the Montefiore or Weiler hospital campuses of Montefiore Medical Center (MMC) in the Bronx, New York. The study time period was from 5/1/2015 to 10/1/2018. All cases of AIS during the study period were identified by a study coordinator via screening daily patient logs and reviewing stroke-related hospital discharges; only index AIS during the study period were included in our analysis. Stroke subtype was defined using TOAST criteria as determined by the treating vascular neurologists at the time of presentation using expert consensus guidelines^7^. For cases where the stroke subtype was not assigned by the treating vascular neurologist, clinical documentation and imaging were used to adjudicate subtypes by two vascular neurologists (CE and ALL). In addition, cryptogenic strokes were further subtyped into embolic strokes of undetermined significance (ESUS) if they had sufficient inpatient work-up to rule-out other etiologies^8^. In order to satisfy the 24 h of cardiac monitoring without atrial fibrillation/flutter required for ESUS designation, we used telemetry monitoring following the stroke during the index admission. All methods were carried out in accordance with relevant guidelines and regulations. Approval for this study and waiver of consent were granted by the Albert Einstein College of Medicine and Montefiore Medical Center institutional review board (IRB # 2018-8832).

### Study variables

For all included patients, we retrospectively collected 40 pre-specified variables of interest via structured review of the electronic medical record (EMR) which included provider care notes, laboratory data, radiology reports, and echocardiograms. We identified comorbid conditions and CVD risk factors (prior stroke/transient ischemic attack [TIA], prior myocardial infarction [MI], diabetes mellitus [DM], hypertension [HTN], tobacco use, heart failure, prior venous thromboembolism [VTE], rheumatic heart disease, human immunodeficiency virus [HIV], atrial fibrillation) via detailed review of emergency and inpatient physician notes^9^. We defined history of hypercoagulability as documentation of a prior VTE or laboratory data of homozygous MTHFR 677C>T, homozygous MTHFR 1298A>C, homozygous or heterozygous factor V Leiden, or homozygous prothrombin G20210A mutations, or presence of anti-phospholipid antibodies. Vital signs and additional laboratory markers of CVD (low-density lipoprotein [LDL], high-density lipoprotein [HDL], triglycerides, and hemoglobin A1c [HbA1c]) were extracted from the EMR. Echocardiography reports from index inpatient hospitalization were manually reviewed to determine measured ejection fraction, left atrial size, and evidence of left ventricular hypertrophy. Radiology reports were used to identify carotid and intracranial atherosclerosis. For the purposes of this study, large vessel atherosclerotic disease was defined as documentation of any vessel stenosis by radiology using any imaging modality during the course of clinical care.

### Statistical analysis

**Figure 1:**
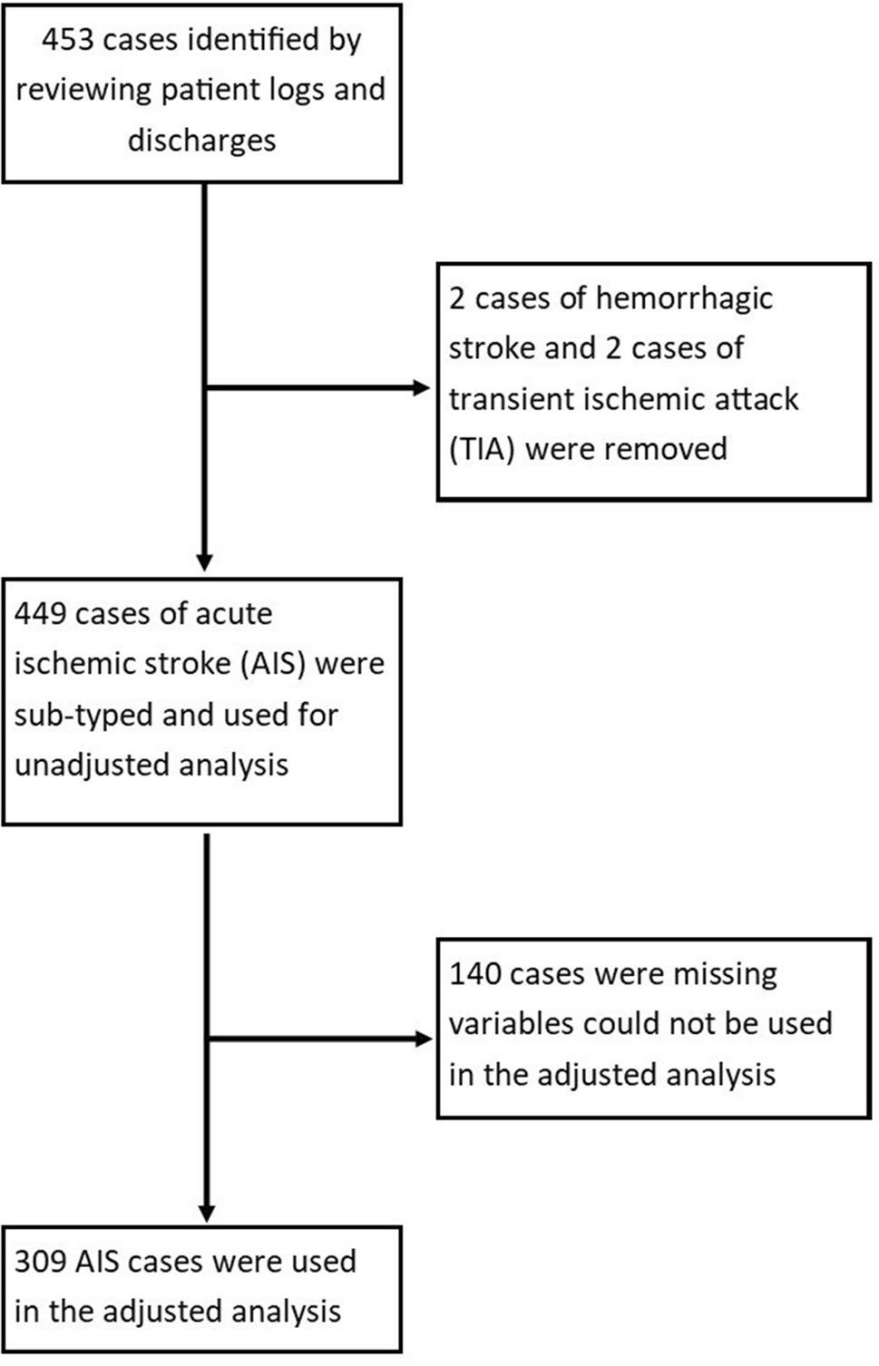
Flowsheet of AIS cases identified in patients aged 18–49 that were used in adjusted and unadjusted analysis.

Categorical variables were compared between different sub-types by χ^2^ analysis. Continuous variables were compared by Student’s T-test. After analyzing unadjusted associations for each determined stroke subtype, we found that, in keeping with prior literature, the small vessel (“lacunar”) and LAA cohorts had near identical CVD phenotypes that were separate from the other subtypes (Supplementary Table 1)^10^. We therefore combined small vessel (“lacunar”) and LAA strokes into a single “vascular” group. Multivariate logistic regression was then used to identify significant independent predictors of either the vascular or CE (termed “cardiac”) subgroup using all available independent clinical factors, biomarkers and imaging findings that were significantly different in the unadjusted analysis. We defined patient’s phenotypic profile as the composite of independently significant risk factors associated with either the vascular or cardiac groups using this regression. Variables for which > 20% of patients were missing values either due to poor documentation in EMR or lack of testing were excluded from this analysis. Clinically non-independent variables were also eliminated from the final model. These included history of DM and admission glucose which were non-independent from HbA1c, left ventricular hypertrophy and left atrial size which were non-independent from ejection fraction, and triglycerides which was non-independent from LDL cholesterol. For the multivariate regression analysis, 140 patients had to be excluded because they were missing values for the included variables (Fig. 1). The final multivariate model was used to characterize cryptogenic stroke cases as fitting either a vascular or cardiac phenotypic profile with > 0.5 probability. Multivariate analysis was done in R; all other analyses were done using Graphpad Prism. Statistical significance was defined with an alpha of < 0.05.

## Results

### General characteristics

We identified a total of 449 young patients with hospital admission for AIS during the period of interest. Of these, there were 145 patients with cryptogenic (32%), 102 with other determined cause (23%), 99 with lacunar (22%), 69 with CE (15%), and 34 with LAA (7.6%) stroke subtypes (Table 1). Of the 145 patients with cryptogenic strokes, 27 (19%) had insufficient work up, 93 (64%) met ESUS criteria and 25 (17%) were considered as having potential competing mechanisms^8^. The mean age across subtypes was 41.1 ± 7.6 and 59.2% were women. Patients with lacunar and LAA strokes were significantly older than patients with CE strokes (mean_lacunar_ = 45.09; mean_LAA_ 44.85; mean_CE_ = 38.5; *p*_*lacunar vs CE*_ < 0.0001; *p*_*LAA vs CE*_ < 0.001). Additionally, a significantly higher proportion of patients with CE strokes were women compared to those with vascular strokes (Table 2). Our population was predominantly made up of race ethnic minorities. There were no differences in race or ethnicity by stroke subtype.

**Table 1:** Population characteristics and biomarker and risk factor profiles for stroke subtypes (n = 449 cases).

Variable	CE n = 69	Cryptogenic n = 145	Small vessel n = 99	LAA n = 34	Other n = 102
Median Age (years) [IQR]	41 [32–46]	42 [36–47]	47 [44–48]	47 [42–49]	40 [32–45]
Female n (%)	44 (63.7)	70 (48.3)	33 (33.3)	13 (38.2)	61 (59.8)
Black n (%) (*% missing*)	28 (43.1) (*10.1*)	57 (42.2) (*14.5*)	42 (42.4) (*7.1*)	13 (38.2) (*2.9*)	33 (33.7) (*6.9*)
Non-Hispanic white n (%) (*% missing*)	3 (4.5) (*4.3*)	8 (6.4) (*14.0*)	10 (10.2) (*1.0*)	3 (8.8)	7 (7.4) (*6.9*)
**Clinical variables**					
Body mass index (mean kg/m^2^ ± SD) (*% missing*)	31.72 ± 8.20 (*4.3*)	31.31 ± 8.12 (*4.1*)	32.33 ± 6.88 (*2.0*)	30.66 ± 6.22 (*2.9*)	29.20 ± 6.09 (*7.8*)
Hypertension (%) (*% missing*)	55.9 (*1.4*)	46.9 (*1.4*)	82.1 (*4.0*)	60.6 (*2.9*)	42.3 (*5.9*)
Diabetes mellitus (%) (*% missing*)	23.5 (*1.4*)	17.5 (*1.4*)	54.7 (*4.0*)	48.5 (*2.9*)	14.3 (*4.9*)
Atrial fibrillation (%) (*% missing*)	18.8	0.0 (*1.4*)	1.0 (*3.0*)	0.0	0.0 (*4.9*)
Ischemic stroke Hx (%) (*% missing*)	17.4	21.0 (*1.4*)	21.1 (*4.0*)	29.4	18.8 (*6.9*)
Congestive heart failure Hx (%) (*% missing*)	26.1	2.80 (*1.4*)	4.2 (*4.0*)	2.9	3.1 (*6.9*)
Rheumatic heart disease Hx (%) (*% missing*)	10.1	0.0 (*1.4*)	1.1 (*4.0*)	0.0	1 (*6.9*)
Myocardial infarction Hx (%) (*% missing*)	7.3	3.5 (*1.4*)	4.2 (*4.0*)	2.9	2.1 (*6.9*)
Clotting Hx (%) (*% missing*)	10.1	4.9 (*1.4*)	2.1 (*4.0*)	0.0	9.4 (*6.9*)
Prior transient ischemic attack (%) (*% missing*)	8.7	13.2 (*0.7*)	11.2 (*1.0*)	11.8	9.7
History of HIV (%) (*% missing*)	5.8	2.8	4.2 (*4.0*)	6.1 (*2.9*)	4.0 (*3.9*)
Family Hx of stroke (%) (*% missing*)	12.9 (*10.1*)	23.9 (*7.6*)	24.2 (*8.1*)	21.2 (*2.9*)	9.3 (*16.7*)
Current tobacco use (%) (*% missing*)	23.5 (*1.4*)	26.4 (*3.4*)	26.3 (*4.0*)	38.2	29.2 (*6.9*)
History of tobacco use (mean pack-years ± SD) (*% missing*)	7.66 ± 9.00 (*44.4*)	15.55 ± 11.26 (*50.0*)	19.22 ± 9.47 (*62.5*)	29.67 ± 17.28 (*62.5*)	15.50 ± 8.99 (*70.6*)
Current cocaine use (%) (*% missing*)	4.4 (*1.4*)	3.6 (*3.4*)	4.3 (*7.1*)	8.8	5.3 (*8.8*)
Systolic blood pressure (mmHg; mean ± SD) (*% missing*)	133.9 ± 23.6 (*24.6*)	140.1 ± 24.5 (*9.0*)	166.4 ± 33.4 (*18.2*)	160.8 ± 37.9 (*26.5*)	138.6 ± 28.0 (*15.7*)
Diastolic blood pressure (mmHg; mean ± SD) (*% missing*)	86.10 ± 19.4 (*24.6*)	84.2 ± 17.0 (*9.0*)	94.4 ± 18.8 (*21.2*)	93.0 ± 15.5 (*38.2*)	84.2 ± 15.4 (*17.6*)
**Imaging variables**					
Carotid atherosclerosis (%) (*% missing*)	3.4 (*15.9*)	3.8 (*9.0*)	2.2 (*9.1*)	20.6	8.0 (*15.7*)
Intracranial atherosclerosis (%) (*% missing*)	20.7 (*15.9*)	42.6 (*11.0*)	33.0 (*5.1*)	73.5	34.4 (*12.7*)
Left atrial size (cm; mean ± SD)	4.0 ± 0.9 (*15.9*)	3.5 ± 0.6 (*15.2*)	3.6 ± 0.6 (*19.2*)	3.6 ± 0.6 (*11.8*)	3.5 ± 0.7 (*20.6*)
Left ventricular hypertrophy (%) (*% missing*)	45.9 (*11.6*)	17.6 (*9.7*)	28.6 (*15.2*)	21.9 (*5.9*)	26.7 (*15.7*)
Ejection fraction (%; mean ± SD) (*% missing*)	43.06 ± 18.54 (*17.4*)	61.04 ± 9.15 (*18.6*)	62.82 ± 6.73 (*17.2*)	63.06 ± 7.25 (*20.6*)	60.13 ± 10.33 (*22.5*)
Patent foramen ovale (%) (*% missing*)	14.0 (*37.7*)	18.7 (*15.2*)	11.9 (*40.4*)	8.0 (*26.5*)	6.6 (*40.2*)
**Laboratory values**					
Admission glucose (mg/dL; mean ± SD) (*% missing*)	130.6 ± 56.8	125.1 ± 64.1 (*0.7*)	195.7 ± 104.1	190.1 ± 108.6 (*2.9*)	121 ± 50.79
Hemoglobin A1c (%; mean ± SD) (*% missing*)	6.7 ± 2.6 (*21.7*)	6.2 ± 1.8 (*5.5*)	8.2 ± 2.6 (*3.0*)	7.8 ± 2.4 (*2.9*)	6.1 ± 1.7 (*23.5*)
Anti-phospholipid antibodies (%) (*% missing*)	12.0 (*63.8*)	5.5 (*24.8*)	0.0 (*72.7*)	0.0 (*58.8*)	6.9 (*43.1*)
Genetic hypercoagulability (%) (*% missing*)	5.6 (*75.4*)	8.8 (*53.1*)	5.6 (*82.8*)	0.0 (*73.5*)	12.5 (*60.8*)
Troponin T (ng/mL; mean ± SD) (*% missing*)	0.060 ± 0.169 (*21.7*)	0.009 ± 0.048 (*22.8*)	0.009 ± 0.035 (*14.1*)	0.011 ± 0.034	0.026 ± 0.109 (*30.4*)
Pro-B-type natriuretic peptide (pg/mL; mean ± SD) (*% missing*)	7885.4 ± 11,052.2 (*71.0*)	1602.7 ± 6547.8 (*80.7*)	542.6 ± 869.5 (*85.9*)	42.5 ± 24.8 (*94.1*)	317.6 ± 749.3 (*84.3*)
Triglycerides (mg/dL; mean ± SD) (*% missing*)	105.9 ± 56.4 (*20.3*)	133.1 ± 80.7 (*4.1*)	190.7 ± 171.1 (*2.0*)	163.1 ± 139.8 (*2.9*)	118.5 ± 64.8 (*23.5*)
Low-density lipoprotein (mg/dL; mean ± SD) (*% missing*)	93.29 ± 38.27 (*20.3*)	99.62 ± 40.55 (*5.5*)	118.2 ± 47.41 (*9.1*)	123.5 ± 55.48 (*8.8*)	90.88 ± 41.86 (*24.5*)
High-density lipoprotein (mg/dL; mean ± SD)	41.44 ± 15.35 (*20.3*)	46.09 ± 15.35 (*4.8*)	42.28 ± 13.19 (*2.0*)	43.39 ± 16.32 (*2.9*)	44.64 ± 16.29 (*24.5*)
Erythrocyte sedimentation rate (mm/h; mean ± SD)	24.80 ± 20.83 (*78.3*)	22.13 ± 22.33 (*51.7*)	32.48 ± 35.79 (*76.8*)	30.88 ± 34.92 (*52.9*)	31.79 ± 27.37 (*52.9*)
C-Reactive protein (mg/L; mean ± SD)	0.48 ± 0.32 (*81.1*)	1.18 ± 2.77 (*55.2*)	1.29 ± 2.56 (*82.8*)	2.36 ± 4.23 (*61.8*)	2.75 ± 6.29 (*60.8*)

**Table 2:** Comparison of biomarkers, risk factors, and imaging findings for small vessel and large artery atherosclerosis vs cardioembolic strokes.

Variable	Small vessel and LAA n = 133	Cardioembolic n = 69	χ^2^ or t-test analysis *p < 0.05 **p < 0.01 ***p < 0.001 ****p < 0.0001
Median age (years) [IQR]	47 [44–48]	41 [32–46]	****
Female n (%)	46 (34.6)	44 (63.7)	****
Black n (%)	55 (41.4)	28 (43.1)	ns
Non-Hispanic white n (%)	13 (9.8)	3 (4.5)	ns
**Clinical variables**			
Body mass index (mean kg/m^2^ ± SD)	31.90 ± 6.74	31.72 ± 8.20	ns
Hypertension (%)	76.6	55.9	**
Diabetes mellitus (%)	53.1	23.5	****
Atrial fibrillation (%)	0.77	18.8	****
Ischemic stroke Hx (%)	23.3	17.4	ns
Congestive heart failure Hx (%)	3.88	26.1	****
Rheumatic heart disease Hx (%)	0.78	10.1	**
Myocardial infarction Hx (%)	3.88	7.3	ns
Clotting Hx (%)	1.55	10.1	**
Prior transient ischemic attack (%)	11.4	8.7	ns
History of HIV	4.6	5.8	ns
Family Hx of stroke (%)	23.4	12.9	ns (*p* < 0.1)
Current tobacco use (%)	29.5	23.5	ns
History of tobacco use (mean pack-years ± SD)	22.2 ± 12.7	7.66 ± 9.00	****
Current cocaine use (%)	5.6	4.4	ns
Systolic blood pressure (mean mmHg ± SD)	165.1 ± 34.5	133.9 ± 23.6	****
Diastolic blood pressure (mean mmHg ± SD)	94.1 ± 18.1	86.10 ± 19.4	*
**Imaging variables**			
Carotid atherosclerosis (%)	7.26	3.4	ns
Intracranial atherosclerosis (%)	43.8	20.7	**
Left atrial size (mean cm ± SD)	3.6 ± 0.6	4.0 ± 0.9	****
Left ventricular hypertrophy (%)	26.7	45.9	*
Ejection fraction (mean % ± SD)	62.88 ± 6.83	43.06 ± 18.54	****
Patent foramen ovale (%)	10.7	14.0	ns
**Laboratory variables**			
Admission glucose (mean mg/dL ± SD)	194.3 ± 104.8	130.6 ± 56.8	****
Hemoglobin A1c (mean % ± SD)	8.1 ± 2.6	6.7 ± 2.6	**
Anti-phospholipid antibodies (%)	0.00	12.0	*
Genetic hypercoagulability (%)	3.85	5.6	ns
Troponin T (mean ng/mL ± SD	0.010 ± 0.035	0.060 ± 0.169	**
Pro-B-type natriuretic peptide (mean pg/mL ± SD)	480.1 ± 827.3	7885.4 ± 11,052.2	*
Triglycerides (mean mg/dL ± SD)	183.7 ± 163.7	105.9 ± 56.4	***
Low-density lipoprotein (mean mg/dL ± SD)	119.50 ± 49.41	93.29 ± 38.27	***
High-density lipoprotein (mean mg/dL ± SD)	42.56 ± 13.99	41.44 ± 15.35	ns
Erythrocyte sedimentation rate (mean mm/h ± SD)	31.82 ± 34.98	24.80 ± 20.83	ns
C-Reactive protein (mean mg/L ± SD)	1.75 ± 3.36	0.48 ± 0.32	ns

### Unadjusted analysis

In the unadjusted analysis, the vascular group (combined lacunar and LAA subtypes) had significantly higher association with hypertension, DM, smoking by pack-years, and intracranial atherosclerosis, and higher systolic blood pressure, diastolic blood pressure, admission glucose, HbA1c, LDL cholesterol, and triglycerides as compared to the CE subgroup (Table 2). The CE subgroup, on the other hand, had a significantly higher association with congestive heart failure, rheumatic heart disease, atrial fibrillation, clinically significant clotting disorders, left ventricular hypertrophy, lower ejection fraction, higher left atrial size, higher pro-BNP and higher mean troponin level as compared to the vascular group (Table 2). There were no significant differences in the presence of patent foramen ovale (PFO) or HDL.

### Adjusted analysis

In the adjusted analysis, we compared the vascular (aggregated lacunar and LAA) and cardiac (CE only) groups using multivariate regression to analyze all the significant clinical factors, biomarkers, and imaging findings from the unadjusted analysis. Only patients that contained all the analyzed variables could be included (n = 309; Fig. 1). After elimination of non-independent variables, our model found six variables associated with the vascular group and five variables associated with the cardiac group (Table 3). For the vascular group, these included age, male sex, hemoglobin A1c, ejection fraction, LDL cholesterol, and family history of AIS. For the cardiac group, these included age, female sex, decreased ejection fraction, CHF history, and lack of intracranial atherosclerosis.

**Table 3:** Significant variables from multivariate regression of vascular (lacunar and LAA) and cardiac (CE) stroke groups.

Variable	Vascular phenotype		Cardiac phenotype	
	OR (95% CI)	*p-value*	OR (95% CI)	*p-value*
Age (years)	**OR = 1.11 (1.03–1.21)**	**0.0107**	**OR = 1.10 (1.00–1.23)**	**0.0818**
Sex (0 = female; 1 = male)	**OR = 5.56 (1.94–11.95)**	**0.0004**	**OR = 0.32 (0.13–1.22)**	**0.0620**
Hemoglobin A1c (%)	**OR = 1.34 (1.08–1.65)**	**0.0086**	OR = 0.85 (0.66–1.13)	0.2882
Ejection fraction (%)	**OR = 1.08 (1.04–1.15)**	**0.0036**	**OR = 0.89 (0.85–0.94)**	**0.00004**
LDL cholesterol (mg/dL)	**OR = 1.02 (1.01–1.03)**	**0.0028**	OR = 1.00 (0.99–1.01)	0.9550
Family Hx of AIS (0 = no; 1 = yes)	**OR = 3.17 (0.97–12.14)**	**0.0713**	OR = 0.57 (0.10–2.55)	0.4774
CHF history (0 = no; 1 = yes)	OR = 0.51 (0.05–5.51)	0.5638	**OR = 14.72 (1.30–244.91)**	**0.0540**
Intracranial atherosclerosis (0 = no; 1 = yes)	OR = 0.97 (0.39–2.64)	0.9536	**OR = 0.10 (0.01–0.52)**	**0.0160**

Bold values reflect variables with *p-values* < 0.1.

**Figure 2:**
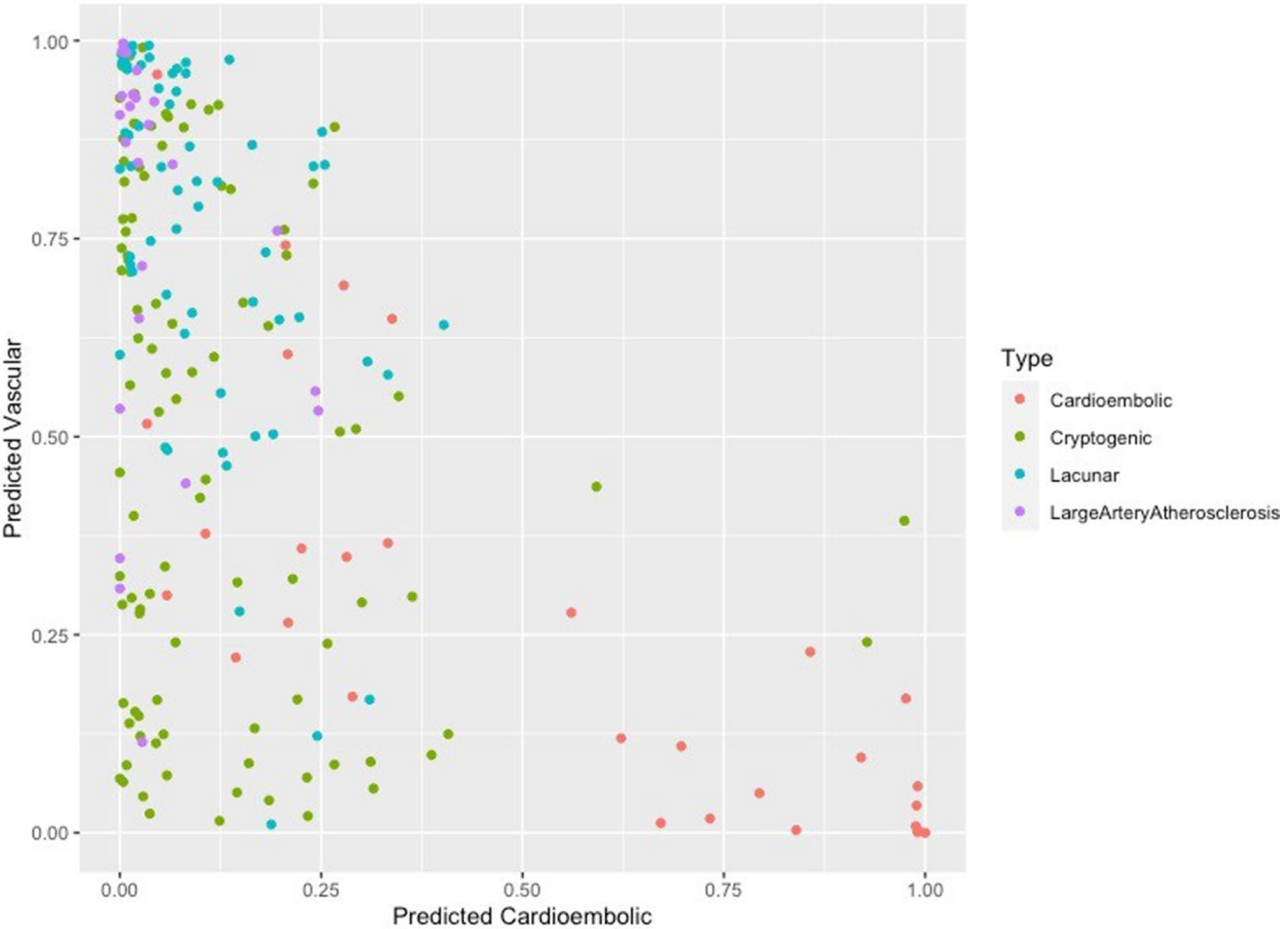
Stroke subtype cases plotted against probability that strokes would be classified as vascular or cardioembolic based on multivariate regression of CVD risk-factor profile.

Finally, we analyzed patients with cryptogenic stroke that had documentation for all of the variables included in the above analysis (n = 97) by fitting them to the risk factor profiles we identified for the vascular and cardiac groups with > 0.5 probability (Fig. 2). This resulted in 50 cases of cryptogenic stroke (51.5%) that fit the vascular phenotype and three cases of cryptogenic stroke that fit the cardiac phenotype (3.1%) with greater than 50% probability. In addition, 45.3% (n = 44 out of 97 cases) of the cryptogenic stroke cases did not fit either of the two defined phenotypes.

## Discussion

AIS in the young is on the rise^2,11^. While some studies show that this trend overlaps with increased prevalence of CVD risk factors, it is not yet clear if CVD accounts for all of the increases observed^2,5^. In our study, the cryptogenic subtype remained the most common etiology of stroke in the young. We confirmed that stroke risk in some of these patients is associated with, and may therefore be driven by, underlying CVD^3,10,12,13^.

It has been reported that there is significant overlap between lacunar and LAA stroke subtypes in CVD risk factors such as HTN, DM, dyslipidemia, and the corresponding biomarkers^9,10^. Similarly, we found that lacunar and LAA stroke subtypes were characterized by a specific CVD risk-factor profile, which we termed the vascular phenotype, while the CE stroke subtype was characterized by a different profile, which we termed the cardiac phenotype. The vascular phenotype was defined by older age, male gender, family history of AIS, and relatively higher HbA1c, EF, and LDL when compared to the cardiac phenotype. The cardiac phenotype was defined by relatively younger age, female gender, history of CHF, absence of intracranial atherosclerosis, and relatively lower EF. When fitting the cryptogenic cases to these two phenotypic models, more than half fit either a vascular or cardiac phenotype. Of these, the majority fit the vascular phenotype.

Our work highlights the opportunity for additional study in the underlying drivers of AIS in the young and suggests that prevention through targeting of traditional CVD risk factors may be of particular importance. Our findings should be contrasted to other studies, which found that cryptogenic stroke was similar to CE stroke, most notably when using profiles of differentially expressed genes^14^. In addition, Embolic Stroke of Undetermined Source (ESUS) is being increasingly recognized as an important subset of cryptogenic stroke^8,15,16^. While our study did not find many cryptogenic strokes that fit the cardiac phenotype, 93 (78.8%) of cryptogenic strokes in our cohort with sufficient work-up met the criteria for ESUS^17,18^. Due to the retrospective nature of our study, the ultimate etiological breakdown for these is not known. The underlying cause for ESUS in young patients is of great interest and an area of future study. Finally, our study cohort was predominantly made up of race-ethnic minorities living in a one of the nation’s poorest urban counties^19,20^. Our findings may therefore differ from previously published findings due to inherent sociodemographic differences in cohorts. To this point, our cohort has a very high burden of vascular risk factors compared to other young stroke cohorts^10,17,21^. A similar study of patients in the Helsinki Young Stroke Registry found that young patients with cryptogenic strokes were more likely to be women and less likely to have vascular risk factors such as HTN and DM^17^. However, in our cohort, many of the cryptogenic stroke cases fit the vascular risk factor phenotype. This suggests that the underlying etiologies of cryptogenic stroke in these cohorts may have different contributing risk factors. It may be that traditional CVD risk factors disproportionately contribute to premature stroke in minorities living in areas of concentrated poverty and, further, that the underlying etiologies of stroke within these populations may be different. This limits the generalizability of our study.

It is also important to note that 45% of the cryptogenic cases did not fit the vascular or cardiac phenotype. This suggests that there are additional risk factors and biomarkers of cryptogenic stroke that are under- or un-recognized. It is also possible, given our missing laboratory data, that the true prevalence of cardiac arrhythmias, hypercoagulable states, and inflammatory conditions were underestimated. Similarly, since carotid webs may be prevalent in 20–25% of patients with cryptogenic stroke but can be difficult to diagnose without dedicated imaging sequences, it is possible that a proportion of our patients were misclassified as having cryptogenic or large artery stroke etiologies when they had unrecognized ipsilateral carotid webs^22,23^.

There are a number of additional limitations of our study. Firstly, this is a retrospective single-center study. Assigned subtypes are therefore prone to misclassification and it could be that patients labeled as cryptogenic strokes in our study were later defined otherwise during further work-up. For this same reason, the retrospective nature of this study prevents further analysis on probable ESUS etiology, limiting our ability to characterize these strokes beyond their subtype classification. Secondly, stroke subtype classification using TOAST is imprecise and, in particular, the lacunar subtype can be biased based on patient’s risk factor profiles as well as variable definitions of lacunar stroke used in clinical practice^7,24–26^. It is unlikely, however, that these known limitations significantly impacted our study outcome given that the proportion of patients in the cryptogenic subgroup was similar to that reported in other cohorts^3,10^. Thirdly, our exclusion of patients with missing data, though necessary for our pre-specified analysis, may have introduced selection bias. Finally, we did not include stroke severity data or detailed imaging characteristics separately in our analyses and instead used stroke etiology as documented by the treating physician limiting our ability to fully characterize the study cohort.

Despite these limitations, similar analytic methods can easily be applied to confirm the validity of our findings given the widespread use of TOAST criteria in clinical practice. We aim for a future where clinical data, biomarkers and imaging findings are all used to calculate the probability of certain risk phenotypes. Separate from the stroke classification system, this would allow for a more patient-centered approach to stroke prevention which could also be made dynamic to account for time-based variations in risk.

## Conclusion

In our cohort, a large proportion of young patients with cryptogenic strokes fit a vascular phenotype of CVD risk based on their clinical, biomarker, and imaging data. Using the CVD phenotype model described herein may inform future stroke prevention strategies.

## Supplementary Information

Supplementary Table 1.

## Data Availability

All the data are available for use by other groups. If you are interested in our data set, please reach out to the corresponding author to begin the process of inter-institutional transfer.

## References

[1] Ovbiagele B (2013). Forecasting the future of stroke in the United States: A policy statement from the American Heart Association and American Stroke Association. Stroke.

[2] George MG, Tong X, Bowman BA (2017). Prevalence of cardiovascular risk factors and strokes in younger adults. JAMA Neurol..

[3] Maaijwee NA, Rutten-Jacobs LC, Schaapsmeerders P, van Dijk EJ, de Leeuw FE (2014). Ischaemic stroke in young adults: Risk factors and long-term consequences. Nat. Rev. Neurol..

[4] Rutten-Jacobs LC (2013). Long-term mortality after stroke among adults aged 18 to 50 years. JAMA.

[5] Otite FO (2017). Increasing prevalence of vascular risk factors in patients with stroke: A call to action. Neurology.

[6] Grau AJ (2001). Risk factors, outcome, and treatment in subtypes of ischemic stroke: The German stroke data bank. Stroke.

[7] Sacco RL (2013). An updated definition of stroke for the 21st century: A statement for healthcare professionals from the American Heart Association/American Stroke Association. Stroke.

[8] Hart RG (2014). Embolic strokes of undetermined source: The case for a new clinical construct. Lancet Neurol..

[9] Boehme AK, Esenwa C, Elkind MS (2017). Stroke risk factors, genetics, and prevention. Circ. Res..

[10] Jaffre A (2014). Risk factor profile by etiological subtype of ischemic stroke in the young. Clin. Neurol. Neurosurg..

[11] George MG, Tong X, Kuklina EV, Labarthe DR (2011). Trends in stroke hospitalizations and associated risk factors among children and young adults, 1995–2008. Ann. Neurol..

[12] Chraa M, Louhab N, Kissani N (2014). Stroke in young adults: About 128 cases. Pan. Afr. Med. J..

[13] Smajlovic D (2015). Strokes in young adults: Epidemiology and prevention. Vasc. Health Risk Manag..

[14] Jickling GC (2012). Prediction of cardioembolic, arterial, and lacunar causes of cryptogenic stroke by gene expression and infarct location. Stroke.

[15] Pirinen J (2020). Left atrial dynamics is altered in young adults with cryptogenic ischemic stroke: A case–control study utilizing advanced echocardiography. J. Am. Heart Assoc..

[16] Mas JL (2001). Recurrent cerebrovascular events associated with patent foramen ovale, atrial septal aneurysm, or both. N. Engl. J. Med..

[17] Martinez-Majander N (2018). Embolic strokes of undetermined source in young adults: Baseline characteristics and long-term outcome. Eur. J. Neurol..

[18] Gratz PP, Gralla J, Mattle HP, Schroth G (2014). Embolic strokes of undetermined source: Support for a new clinical construct. Lancet Neurol..

[19] Bureau, U. S. C. QuickFacts Bronx County (Bronx Borough), New York (2019).

[20] Bleiwas KB (2014). An Economic Snapshot of the Bronx.

[21] Rolfs A (2013). Acute cerebrovascular disease in the young: The Stroke in Young Fabry Patients study. Stroke.

[22] Sajedi PI (2017). Carotid bulb webs as a cause of "Cryptogenic" ischemic stroke. AJNR Am. J. Neuroradiol..

[23] Kim SJ (2019). Carotid webs in cryptogenic ischemic strokes: A matched case–control study. J. Stroke Cerebrovasc. Dis..

[24] Meschia JF (2006). Interobserver agreement in the trial of org 10172 in acute stroke treatment classification of stroke based on retrospective medical record review. J. Stroke Cerebrovasc. Dis..

[25] Jackson C, Sudlow C (2005). Are lacunar strokes really different? A systematic review of differences in risk factor profiles between lacunar and nonlacunar infarcts. Stroke.

[26] Shi Y, Wardlaw JM (2016). Update on cerebral small vessel disease: A dynamic whole-brain disease. Stroke Vasc. Neurol..

