# Supplementary material for: Associating cryptogenic ischemic stroke in the young with cardiovascular risk factor phenotypes
Source: Sci Rep. 2021 Jan 11;11:275. doi: 10.1038/s41598-020-79499-1 (PMC7801422; doi:10.1038/s41598-020-79499-1)
Supplement: Supplementary file 1 — Supplementary Table 1. [file 41598_2020_79499_MOESM1_ESM.docx]

**Supplementary Data**

**Title**: Associating Cryptogenic Ischemic Stroke in the Young with Cardiovascular Risk Factor Phenotypes

**Authors:**

Joseph M. Dardick, BA,^1*^ David Flomenbaum, BA,^1^ Daniel Labovitz, MD MS,^1,2^ Natalie Cheng, MD,^1,2^ Ava L. Liberman, MD,^1,2†^ and Charles Esenwa, MD MS^1,2†^

^1^ Department of Neurology, Albert Einstein College of Medicine, Bronx, New York

^2^ Stern Stroke Center, Montefiore Medical Center, Bronx, New York

^†^ Co-senior authors

* Corresponding Author: Joseph M. Dardick

Phone # – 314-249-4648

Email – joseph.dardick@einsteinmed.org

Address – 3316 Rochambeau Avenue, Bronx, NY 10467

**Supplementary Table 1.** Comparison of Biomarkers, Risk Factors, and Imaging Findings for Small Vessel vs Large Artery Atherosclerosis

|  | Small Vessel  n=99 | LAA n=34 | χ^2^ or t-test analysis |
| --- | --- | --- | --- |
| Median Age (years) [IQR] | 47 [44 – 48] | 47 [42 – 49] | ns |
| Female n (%) | 33 (33·3) | 13 (38·2) | ns |
| Black n (%) | 42 (42·4) | 13 (38·2) | ns |
| Non-Hispanic white n (%) | 10 (10·2) | 3 (8·8) | ns |
| Clinical Variables |  |  |  |
| Body Mass Index (mean kg/m^2^ ± SD) | 32·33 ± 6·88 | 30·66 ± 6·22 | ns |
| **Hypertension (%)** | **82·1** | **60·6** | ***** |
| Diabetes Mellitus (%) | 54·7 | 48·5 | ns |
| Atrial Fibrillation (%) | 1·0 | 0·0 | ns |
| Ischemic Stroke Hx  (%) | 21·1 | 29·4 | ns |
| Congestive Heart Failure Hx (%) | 4·2 | 2·9 | ns |
| Rheumatic Heart Disease Hx (%) | 1·1 | 0·0 | ns |
| Myocardial Infarction Hx (%) | 4·2 | 2·9 | ns |
| Clotting Hx (%) | 2·1 | 0·0 | ns |
| Prior Transient Ischemic Attack (%) | 11·2 | 11·8 | ns |
| History of HIV | 4·2 | 6·1 | ns |
| Family Hx of Stroke (%) | 24·2 | 21·2 | ns |
| Current Tobacco Use (%) | 26·3 | 38·2 | ns |
| History of tobacco use (mean pack-years ± SD) | 19·22 ± 9·47 | 29·67 ± 17·28 | ns |
| Current Cocaine Use (%) | 4·3 | 8·8 | ns |
| Systolic blood pressure (mean mmHg ± SD) | 166·4 ± 33·4 | 160·8 ± 37·9 | ns |
| Diastolic blood pressure (mean mmHg ± SD) | 94·4 ± 18·8 | 93·0 ± 15·5 | ns |
| Imaging Variables |  |  |  |
| **Carotid Atherosclerosis (%)** | **2·2** | **20·6** | ******* |
| **Intracranial Atherosclerosis (%)** | **33·0** | **73·5** | ******** |
| Left Atrial Size (mean cm ± SD) | 3·6 ± 0·6 | 3·6 ± 0·6 | ns |
| Left Ventricular Hypertrophy (%) | 28·6 | 21·9 | ns |
| Ejection Fraction (mean % ± SD) | 62·82 ± 6·73 | 63·06 ± 7·25 | ns |
| Patent Foramen Ovale (%) | 11·9 | 8·0 | ns |
| Laboratory Variables |  |  |  |
| Admission Glucose (mean mg/dL ± SD) | 195·7 ± 104·1 | 190·1 ± 108·6 | ns |
| Hemoglobin A1c (mean % ± SD) | 8·2 ± 2·6 | 7·8 ± 2·4 | ns |
| Anti-phospholipid antibodies (%) | 0·0 | 0·0 | n/a |
| Genetic hypercoagulability (%) | 5·6 | 0·0 | ns |
| Troponin T (mean ng/mL ± SD | 0·009 ± 0·035 | 0·011 ± 0·034 | ns |
| Pro-B-type Natriuretic Peptide (mean pg/mL ± SD) | 542·6 ± 869·5 | 42·5 ± 24·8 | ns |
| Triglycerides (mean mg/dL ± SD) | 190·7 ± 171·1 | 163·1 ± 139·8 | ns |
| Low-density lipoprotein (mean mg/dL ± SD) | 118·2 ± 47·41 | 123·5 ± 55·48 | ns |
| High-density lipoprotein (mean mg/dL ± SD) | 42·28 ± 13·19 | 43·39 ± 16·32 | ns |
| Erythrocyte Sedimentation Rate (mean mm/hr ± SD) | 32·48 ± 35·79 | 30·88 ± 34·92 | ns |
| C-Reactive Protein (mean mg/L ± SD) | 1·29 ± 2·56 | 2·36 ± 4·23 | ns |
